# Awareness and Knowledge Levels of Cervical Cancer and HPV and Attitudes Toward HPV Vaccination in Algeria

**DOI:** 10.3390/vaccines14080676

**Published:** 2026-08-05

**Authors:** Mohamed Lounis, Djihad Bencherit, Mohamed Abed, Zahia Ben Aissa, Zahia Gaafazi

**Affiliations:** 1 Department of Agro-Veterinary Sciences, Faculty of Natural and Life Sciences, University of Djelfa, P.O. Box 3117, Djelfa 17000, Algeria; 2 Laboratoire d’Exploration et Valorisation des Écosystèmes Steppiques, Faculty of Natural and Life Sciences, University of Djelfa, P.O. Box 3117, Djelfa 17000, Algeria; djihad.bencherit@univ-djelfa.dz; 3 Department of Biology, Faculty of Natural and Life Sciences, University of Djelfa, P.O. Box 3117, Djelfa 17000, Algeria

**Keywords:** cervical cancer, screening, HPV, HPV vaccine, hesitancy, Algeria

## Abstract

Background/Objectives: Cervical cancer (CC), mainly caused by human papillomavirus (HPV), is one of the major cancers threatening women’s health in Algeria. However, despite the existence of a cost-effective vaccine, it has not yet been introduced into the national vaccination program. Recent information indicated that its introduction will be imminent. Thus, the current study was conducted to evaluate the level of awareness and knowledge about CC and HPV among the Algerian population as well as its attitudes toward HPV vaccination. Methods: A cross-sectional survey was carried out between 4 November 2022 and 8 May 2023 using a self-administered questionnaire that was diffused through social media platforms. Results: A total of 414 participants were included in our sample, with the dominance of those aged 18 to 30 years old (74.6%), those with university education (75.8%), and those with a medium financial level (77.5%). A high level of awareness was reported (76.3%), especially among healthcare workers (98.4%, OR: 17.099, 95% CI: 2.294–127.476, sig. = 0.06), participants aged more than 30 years (87.6%, OR: 2.78, 95% CI: 1.313–5.883, sig. = 0.008), and females (85.1%, OR: 2.02, 95% CI: 1.15–3.548, sig. = 0.014). However, the level of knowledge did not follow, since less than a third (32.1%) of the participants only were aware of HPV, and multiple gaps were reported regarding its epidemiology and its prevention. Moreover, while 30.8% of the participants were willing to get the HPV vaccine, 40.5% were hesitant, and 27.7% were resistant. The causes of rejection/hesitancy included mainly complacency, vaccine refusal, and the lack of confidence in the vaccine. In addition, hesitant and reluctant participants have shown a low level of knowledge and a high score of conspiracy beliefs. Conclusions: These results provide new insights into knowledge and attitudes towards CC and HPV vaccination. It reported that despite the high level of awareness about CC, gaps of knowledge remain and vaccination hesitancy/reluctance is persistent. These results suggest the need for raising awareness particularly about HPV which will consequently help to increase HPV vaccine acceptance.

## 1. Introduction

Cancers are the second leading cause of mortality around the world, responsible for more than 10 million, or nearly 20%, of all deaths in 2023 [1]. It has been estimated that nearly 40% of all new cancer cases in 2022 were linked to preventable causes [2]. These causes comprised around 30 factors, including, among others, smoking, alcohol consumption, 13 occupational exposures, and nine infectious agents [2,3]. Lung, stomach and cervical cancers represented nearly half of all preventable cases in both men and women [2,3]. The last one, cervical cancer (CC), is the fourth most common cancer in women globally in 2023, with an estimated number of 886,538 diagnosed women, and is among the top ten in terms of mortality, with about 369,397 deaths [4]. Most of these cases and deaths are reported in low- and middle-income countries [5].

CC is overwhelmingly caused by oncogenic types of human papillomavirus (HPV), a common sexually transmitted virus with a cutaneous and mucosal tropism that could affect the skin, throat, and anal and genital areas [6]. Although HPV infects silently almost all sexually active people at some point in their lives, its persistence could cause genital warts and cancers, mainly cervical cancer in women and other less common cancers in different organs in the oropharyngeal, the anal and the genital areas in both genders [7].

Fortunately, CC is considered a highly preventable disease [6]. This could be done through HPV vaccination and early screening and treatment of precancerous lesions [8].

Vaccination is one of the most cost-effective tools to prevent CC and HPV-related cancers. Several HPV vaccines have been licensed worldwide. However, only five of them are currently prequalified by the WHO [6,9]. All these vaccines protect against highly pathogenic HPV types responsible for more than three-quarters of CC cases [6]. These vaccines, which have proved their safety, are in priority recommended for all girls aged 9–14 years, before their sexual debut. Some countries have additionally included boys in their HPV vaccination programs to further decrease HPV circulation in the population and prevent HPV-associated cancers among males [6]. Currently, more than 150 countries have included the HPV vaccine in their national immunization program [10]. However, vaccination rates remain low, particularly in low- and middle-income countries [6,11,12]. These rates are far from the 90% vaccine coverage target by the year 2030 fixed in the WHO global strategy to accelerate the elimination of cervical cancer (WHO, 2020 global). The other targets of this strategy are to achieve the screening of 70% of women by the age of 35 years, and again at 45 years old, and to treat 90% of women with cervical precancer or cancer [13]. If this goal is met in 2030, more than 300,000 deaths might be averted in the same year, over 14 million by 2070, and over 62 million by 2120 [14].

To achieve this goal, focus should remain not only on ensuring vaccine accessibility and expanding screening and treatment facilities but also on increasing awareness and knowledge about CC, HPV infection, vaccination, and CC early screening [15,16]. In fact, awareness, knowledge and conspiracy beliefs are intimately associated with vaccine and screening uptake [17,18,19,20,21,22]. In this way, several studies were undertaken to evaluate the level of knowledge, attitude, and practice throughout the world [22,23,24,25,26,27], even before the introduction of the vaccine. This type of survey is crucial for identifying who is unprepared, what misinformation is circulating, which messengers matter most, and how to tailor education and communication before rollout begins. This is the case in Algeria, where the vaccine is not yet introduced, even though CC is ranked fourth among female cancers with more than 1500 cases annually and 15.2 million women above 15 years old at risk of developing CC [28,29]. Without vaccination, the number of deaths is estimated to rise by more than 95% by 2040 compared to 2020 [30]. The prevention strategy in the country is mainly based on early screening, officially recommended since 2003 for women aged 30 to 65 years at a frequency of 3 years [30]. However, 70% of CC cases are detected late in Algeria [29]. These data suggest a lack of awareness and knowledge about CC and HPV and low adherence to early screening [22]. Thus, raising awareness and knowledge about CC and HPV may aid in the adoption of more positive attitudes and behaviors around vaccination and early screening.

In this way, this study was conducted to evaluate the level of awareness and knowledge about CC and HPV among Algerians and their attitudes toward HPV vaccination shortly before its rollout.

## 2. Materials and Methods

### 2.1. Study Design

A cross-sectional online survey was conducted between 4 November 2022 and 8 May 2023, targeting all Algerians aged 18 years or older, and currently living in the country. The questionnaire that was developed in Arabic and French languages was disseminated through social media platforms including WhatsApp, Viber, and Facebook. Participants were invited to complete the questionnaire through a Google Forms link. The study was conducted following the Strengthening of the Reporting of Observational Studies in Epidemiology (STROBE) guidelines for cross-sectional studies [31]. Participation was voluntary and without any incentives. Participants were allowed to participate after providing their consent and were able to leave the survey at any stage. They were assured that the analysis was conducted confidentially and that any personal identifiable information was requested.

The surveyMonkey online sample size calculator [32] was used to compute the required minimum sample size. The figure was 385 based on the following formula for an infinite population (*N* = 45,477,389 Algerians for the year 2022)

$$n=Z^{2}\frac{pq}{e^{2}}$$

with the following assumptions:

*n* is the minimum sample size (here, *n* = 385);
*Z*^2^ = 1.96 for α = 0.05 (confidence interval of 95%);
*p* is the proportion (*p* = 0.5);
*q* = 1 − *p*;
*e* is the accepted margin of error (ME) (here, we chose an ME of 5%, or 0.05).

### 2.2. Survey Instrument

The questionnaire for this study was constructed based on prior KAP surveys addressing HPV and CC [22,23,24]. It was divided into multiple sections: The first part contains a short introduction describing the objectives of the study and requiring participants’ consent. After providing their consent, participants were first asked about their sociodemographic characteristics including age, educational level, gender, marital status, area of residence, financial level, and whether they were healthcare workers.

They were then asked whether they had heard of CC, their sources of information, their knowledge of its causes, their awareness of its early screening, their statuses toward CC screening and the barriers to its uptake.

The following section sought to describe the level of awareness of the participants about HPV, their sources of information, their levels of knowledge and their attitudes toward HPV vaccines and the potential barriers causing reluctance. Lastly, vaccine beliefs were assessed using the Vaccine Conspiracy Beliefs Scale (VCBS) to evaluate potential relationships with HPV vaccine attitudes [33].

### 2.3. Data Management and Measures

The level of knowledge was assessed using 11 items with “yes”, “no”, or “I do not know” responses (acceptable internal consistency (Cronbach’ alpha = 0.725)). The score of knowledge was calculated by attributing points of 1 for each correct response and 0 for incorrect responses. Responses of “I do not know” were given a score of 0. The total score, ranging from 0 to 11, was calculated for each individual, and the mean score (±SD) was calculated by dividing the sum of scores by the number of respondents. This mean score was used to determine whether a difference exists between the different demographic variables (age, gender, education, marital status, financial level, residence and health professions status), and the attitude toward the HPV vaccine.

Regarding the Vaccine Conspiracy Beliefs Scale (VCBS), the seven-item previously validated scale was used. The scale demonstrated high internal consistency (Cronbach’s alpha = 0.937). Each item was rated on a seven-point Likert scale ranging from 1 for “strongly disagree” to 7 for “strongly agree” with higher scores indicating higher conspiracy beliefs. The mean VCBS (±SD) was calculated for the overall population and according to participants’ attitudes toward the HPV vaccine (willingness, hesitancy, and reluctance).

### 2.4. Statistical Analysis

Excel 2007 and SPSS version 22 were used to analyze the results. Descriptive analyses were first conducted to calculate numbers and frequencies classifying the participants according to their demographic characteristics. They were also used to calculate the level of awareness and the frequencies of correct/incorrect responses of items related to knowledge of different chapters of CC and HPV. Means (±SD) were also calculated for the knowledge and the VCBS scores.

Bivariate analyses were conducted using Chi-squared and Fisher tests to compare proportions while *t*-tests and one-way ANOVA were used to compare means.

Significant results were subjected to logistic regression analysis to identify the factors that were associated with the level of awareness of CC and HPV. All statistical analyses were done considering a significance (sig.) of 0.05.

## 3. Results

### 3.1. Demographic Characteristics

A total of 442 individuals answered the questionnaire. After excluding incomplete or suspected careless responses and those with a lack of e-consent, 414 questionnaires were subject to further analysis.

The majority of respondents were aged 18 to 30 years old (74.6%), had university education (75.8%) and reported a medium financial status (77.5%). Participants living in urban areas accounted for 93% of the study population, while males represented more than three-fifths (62.8%) of the respondents, and seven of ten participants were single (71.7%). Finally, healthcare workers constituted only 15.2% of the study population (Table 1).

### 3.2. Cervical Cancer Awareness and Knowledge

The results showed that, out of the 414 respondents, 76.3% declared that they had heard of CC before this study. The rate of awareness varied from 57.4% (among individuals with a low financial level) to 98.4% (among healthcare workers). High rates were also reported among participants aged more than 30 years (87.6%), females (85.1%), and married individuals (84.6%), and these results were significantly different from their counterparts with *p*-values of 0.002 for age and gender, 0.018 for marital status, 0.004 for financial level, and <0.001 for healthcare profession status (Table S1, Supplementary Materials).

The results of the logistic regression analysis showed that participants of more than 30 years old (OR: 2.78, 95% CI: 1.313–5.883, sig. = 0.008), females (OR: 2.02, 95% CI: 1.15–3.548, sig. = 0.014), participants reporting a medium financial status level (OR: 2.252, 95% CI: 1.149–4.413, sig. = 0.018), and healthcare workers (OR: 17.099, 95% CI: 2.294–127.476, sig. = 0.06) showed higher odds of awareness of CC than their counterparts (Table 2).

**Figure 1:**
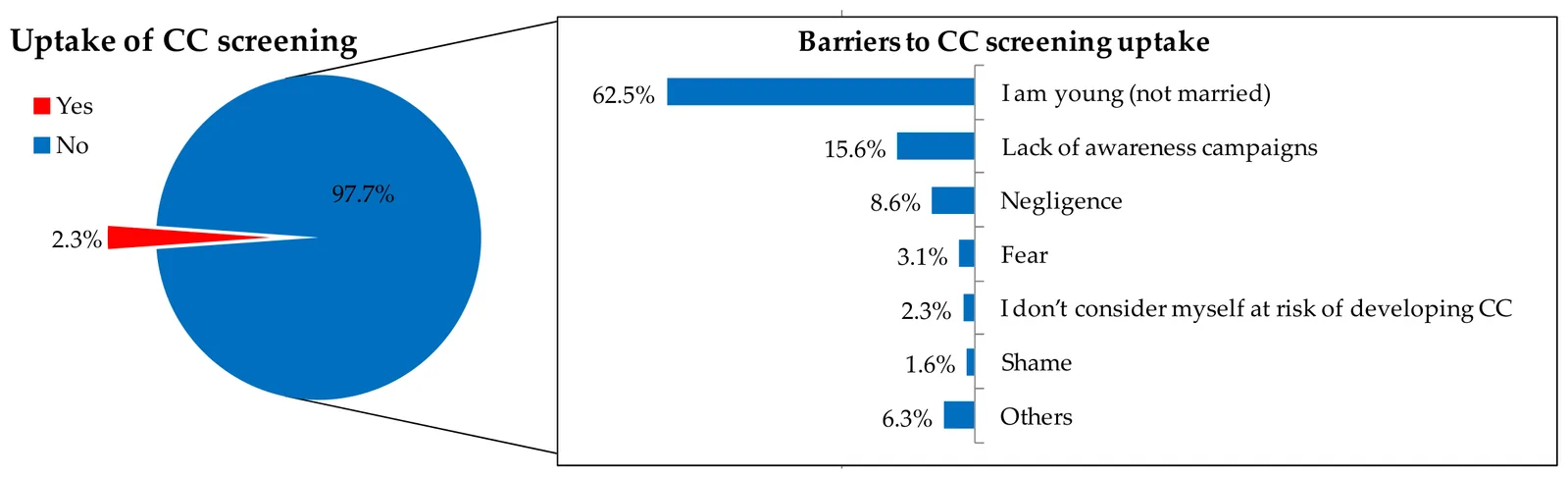
Screening uptake and its barriers.

Regarding their knowledge, slightly more than half of the participants who had heard of CC, 50.9% declared knowing its cause, and 30.9% mentioned HPV. Furthermore, 58.2% of these participants were aware of CC early screening, and nearly all females reported not having undergone this test. Being young and/or single (62.5%), the lack of awareness campaigns (15.63%), and carelessness (8.6%) were the most important causes of this poor screening uptake (Figure 1).

**Figure 2:**
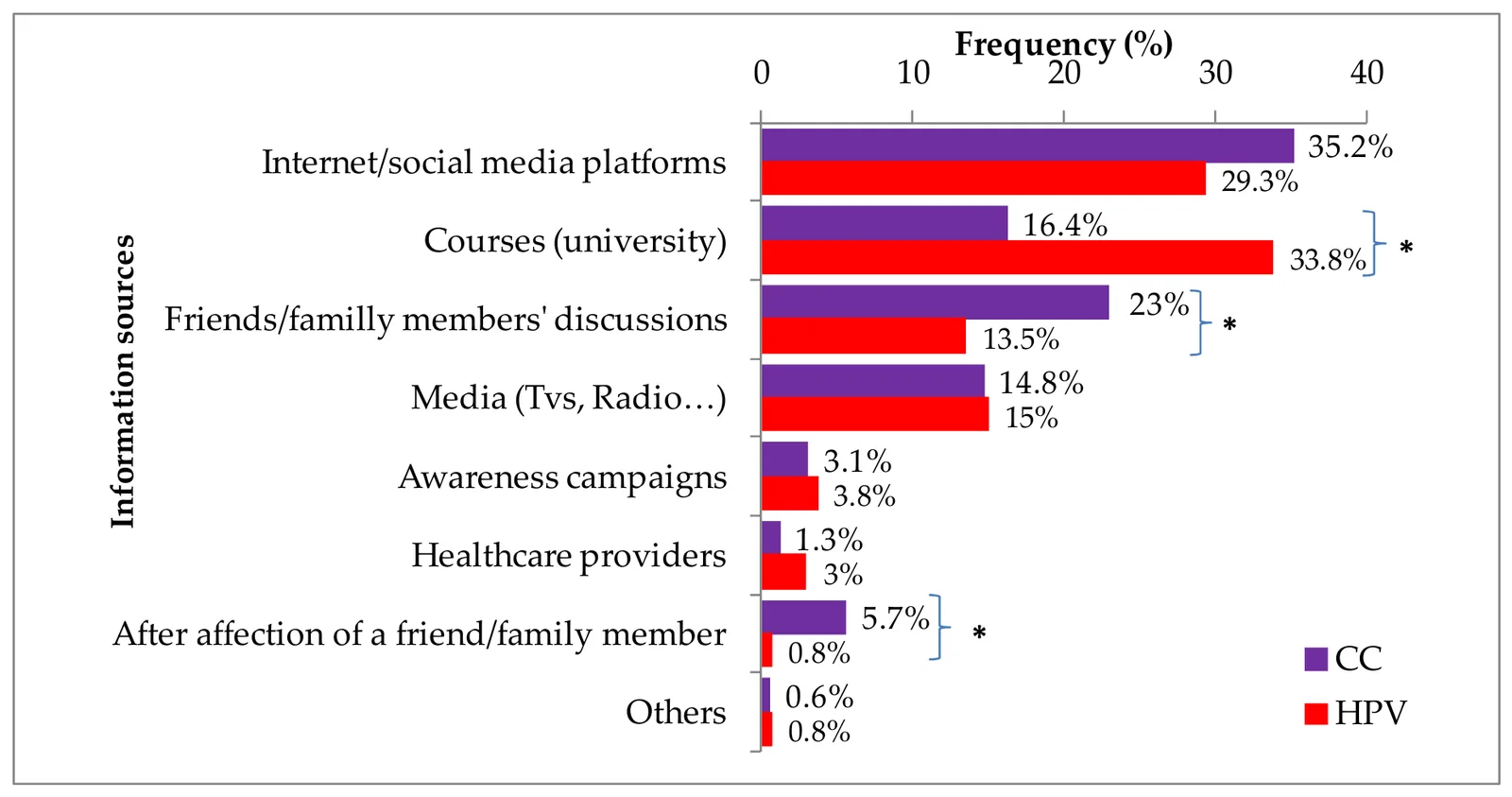
Source of information about CC and HPV of the study population. (* indicated significant difference between CC and HPV).

Finally, the sources of information of the participants who had heard of CC were mainly represented by the Internet and social media platforms (35.2%), courses (16.4%), friends’ and family members’ discussions (23%), and mass media (14.3%) (Figure 2).

### 3.3. HPV Awareness and Knowledge

The results of this study showed that more than two-thirds of the participants (67.9%) have not ever heard of HPV. Healthcare workers (79.4%) and those with a high financial level (50%) showed the highest rate of awareness whereas those with low incomes (17%) and those living in rural areas (17.2%) were less aware of this virus (Table S2, Supplementary Materials).

The rate of awareness differed substantially by age (sig. = 0.025), gender (0.022), financial status (sig. = 0.003), and healthcare profession (sig. < 0.001). These results were supported by logistic regression analysis of age (OR (over 30 years old/18–30 years old): 2.175, 95% CI: 1.272–3.719, sig. = 0.005), financial level (OR (high/low): 3.189, 95% CI: 1.162–8.751, sig. = 0.024), and health profession (OR (healthcare worker/non-healthcare workers): 12.591, 95% CI: 6.177–25.661, sig < 0.001) (Table 3).

For their sources of information about HPV, the respondents cited mainly courses (33.8%), the Internet/social media platforms (29.4%), mass media (15%), and friends’ and family members’ discussions (13.5%) (Figure 2).

Regarding the level of knowledge, the results showed a medium of correct responses of 65.4%, corresponding to a mean score of 6.54 (SD = 2.832).

**Figure 3:**
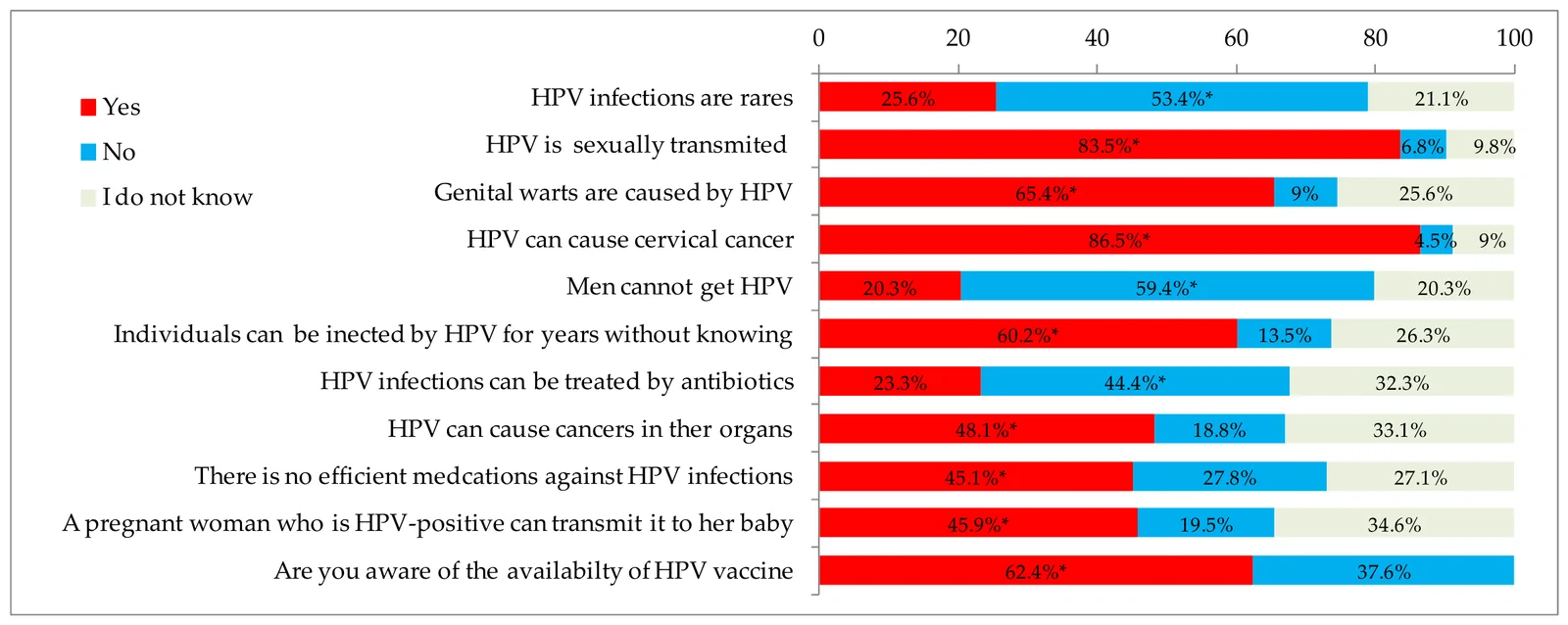
Responses of the participants to the questions evaluating HPV knowledge. (* indicated correct responses).

The highest rates of correct responses were obtained for the items “*HPV can cause cervical cancer*” (86.5%) and “*HPV is sexually transmitted*” (83.5%), and the lowest rates were obtained for the items “*HPV infection can be cured by antibiotics*” (44.4%) and “*There is no efficient medication against HPV*” (45.1%) (Figure 3).

The score of knowledge was significantly higher among participants with university education (sig. = 0.023), those living in urban areas (sig. = 0.04) and healthcare workers (sig. < 0.001) (Table 4).

### 3.4. HPV Vaccine Attitudes

The results showed that 62.4% were aware of the availability of the HPV vaccine, whereas only 2.3% had received it. Furthermore, 30.8% of the participants were willing to receive the vaccine, while 41.5% were hesitant, and 27.7% were reluctant. The most important factors of vaccine hesitancy or reluctance were “*I do not consider myself at risk of HPV infection*” (35.6%), “*I do not consider HPV as a common infection in Algeria*” (17.4%), and “*Visiting a doctor makes me uncomfortable, which keep me from vaccination*” (12.8%) (Table 5).

Finally, attitudes toward HPV vaccination were significantly associated with the score of knowledge and the VCBS scores (Table 6).

## 4. Discussion

This study provides up-to-date information regarding awareness, knowledge, and attitudes on CC and HPV in a sample of the Algerian population. The results revealed a high level of awareness of CC (76.3%). The rate of awareness was expectedly higher among females (85.1%) than males (71.1%). The same observation was reported by Mbulawa et al. [34] in South Africa and El Mansouri et al. [35] in Morocco. The rate of awareness reported among females aligns with the rate observed among female students reported previously in Algeria (88.4%%) [22], and also with the rates obtained among females in Kenya (86%) [27], in Kuwait (85.8%) [36], and among female university students in Morocco (81.1–89%) [35,37]. However, the rate of awareness could vary substantially among females according to their sociodemographic characteristics, including age, marital status, residence, and profession [38,39,40,41,42,43,44,45,46].

In contrast to the results of previous studies in Algeria [22,47], 58.2% were aware of its early detection, while nearly all female participants declared having not undergone CC screening. By omitting young age, lack of awareness was the most common barrier cited by the study participants. In the same way, a systematic review conducted in low- and middle-income countries found that the most important barrier to CC screening uptake is the lack of information and education about CC, CC screening, and prevention [48]. More intriguingly, a study in Tanzania revealed that 70.3% of university students did not think that screening helps to prevent CC [49]. In addition, a study conducted in the Eastern Cape Province rural community in South Africa revealed that women previously screened for CC had a significantly higher cervical knowledge score than those who had never been screened [34]. It is worth noting that one of the WHO objectives to eliminate CC and sexually transmitted diseases is to increase the percentage of women being screened to 70% by 2030 [13].

On the other hand, about half, 50.9%, of the participants who had heard of CC declared knowing its cause, and the majority of them incriminated HPV. Later, 115 out of the 133 students who had heard of HPV (86.5%) were aware that this virus can cause CC. Except for certain populations, including mainly medical students [23], the lack of knowledge about the link between CC and HPV is common. If this information was shared by half of female staff in a Nigerian university (50%) [46] and more than four of ten women in South Africa (43.2%) [34], low rates were reported among rural (22.9%) and urban (24.4%) women in India [45], young women of Dhulikhel municipality in Nepal (20.4%) [42], women in hard-to-reach areas of Bangladesh and their family decision-makers (~20%) [41], and women in the Caribbean indigenous community (17%) [40]. A recent systematic review reported that only 14% of Arab women knew that HPV causes CC [50]. More intriguingly, a recent study in Morocco revealed that only 10% of parents of young girls knew that cervical cancer is caused by a persistent HPV infection [51].

Of note, less than a third of the participants (32.1%) of our study declared having heard of HPV. The same rate of awareness was reported among women of the western region in Saudi Arabia (34.6%) [25], while higher rates were reported among women of reproductive age in Kiambu County, Kenya (44.7%) [27], and Kuwaiti female students (55.3%) [24]. A much higher rate of awareness of HPV was reported in the United States of America (USA), ranging from 81.8% to 95.3% among women [52] and college students [53], respectively. These high rates may certainly be related to the multiple public health campaigns designed to promote the benefits of HPV vaccination to parents and adolescents in this country [53]. This supports the idea that there is a need for awareness campaigns for the Algerian population about this infectious agent.

Higher rates were also reported among medical students in some Arab countries, including Jordan (72%) [23], Saudi Arabia (91.7%) [54], and the United Arab Emirates (97%) [55], while the rate was very low among students in the neighboring country of Morocco (10 to 14.7%) [35,37].

In our study, healthcare workers, older individuals (over 30 years old), and those with a high financial level showed the highest rates of awareness of HPV, while female gender was not a significant predictor. In a previous study among Algerian students, females were nearly two times as likely to have heard of HPV as males (OR = 1.7, 95% CI = 1.161–2.489) [22]. Regarding age, a study conducted in the western region of Saudi Arabia reported that women aged more than 40 years were less aware of HPV than younger women [25]. The same observation was reported among female caregivers in the USA, where younger ones aged 21–50 were nearly 2.5 times more likely to have heard of HPV than their counterparts (OR = 2.47, 95% CI = 1.49–4.08) [26].

In addition to the low rate of awareness about HPV reported in this survey, multiple gaps of knowledge were found among those who heard of this pathogen. Particularly, more than half of these participants were not aware that HPV can cause cancer in organs other than cervix, that HPV infection cannot be cured with medicines, and that antibiotics are not effective against HPV. Similar gaps of knowledge were previously reported among students in Algeria [22], and in other countries [23,24]. However, the striking result is the fact that nearly four of ten of those who had heard of HPV were not aware of the availability of the HPV vaccine. Even though the rate of awareness is higher than the rate reported by Bencherit et al. [22] among university students in our country, this rate remains very low when adjusted to the total number of participants in our sample. In general, awareness of HPV vaccine varied substantially, with developed countries showing the highest rates of awareness [37,51,53]. More interestingly, high proportions of participants were not aware of the national program to prevent CC and HPV infections in some low-and middle-income countries [49,51].

Not surprisingly, all but three of the participants who had heard of HPV in our study population were unvaccinated. This finding is a consequence of the absence of the HPV vaccine in the national immunization program and the lack of public awareness campaigns to promote its acceptability. However, nearly a third of these students were willing to get the vaccine if available (30.8%). This rate is higher than the rate reported among female students in our country (26.7%) [22], but remains lower than the rates reported in other African and Arabic countries including Morocco (60–67%) [37,51], Kuwait (57.4–69.8%) [24,36], the United Arab Emirates (74%) [55], Saudi Arabia (75.8%), [25] and Jordan (75%) [23]. The low rate of vaccine acceptance remains distant from the WHO 90-70-90 goal of 90% vaccinated women by 2030 [13].

This low rate of acceptance could certainly be related to the lack of education regarding CC, HPV, and HPV vaccination. The role of awareness campaigns and healthcare providers was very limited in public awareness in our study. In fact, targeted awareness campaigns and healthcare providers’ recommendations are considered key factors for vaccine acceptance and uptake [56,57]. A recent meta-analysis reported that provider recommendation increased vaccination uptake from 24% to 60% [58]. Unfortunately, the role of healthcare providers in the spreading of information tends to decrease in favor of social media platforms in multiple countries, including Algeria [22,25,50]. This shift towards informal sources of information is concerning, given the documented proliferation of misinformation about vaccine safety and efficacy on social media platforms, thus reinforcing the endorsement of conspiracy theories [50]. In our study, conspiracy beliefs were significantly associated with vaccine reluctance. Similar results were previously reported, especially among students in Algeria and in other Arab countries [22,23,24,50].

In addition, complacency, which refers to the underestimation of disease risk and doubts regarding the necessity of vaccination, was the most important factor contributing to HPV vaccine reluctance and hesitancy in our study. The same observation was reported among students in Algeria [22], female students in Kuwait [24], medical students in Jordan [23], and women in Saudi Arabia [25]. A recent study among adolescents in China revealed that high complacency, low confidence, and low HPV knowledge were significant determinants of HPV vaccine hesitancy [59].

Finally, this study has shown some limitations that could affect the quality of its results. The cross-sectional online design could be subject to multiple biases. The cross-sectional nature of this survey allows it to provide information about the period from study that may change over time. The online nature did not allow for obtaining a representative sample since it can marginalize some categories, including those with limited access to the Internet and older individuals who are generally less connected to social media than younger ones. Furthermore, the self-administered questionnaire nature of this survey could be subject to social desirability biases. Finally, due to the country’s epidemiological context and national health policy, which plans to introduce HPV vaccination primarily for cervical cancer prevention, our study focused exclusively on CC, without considering other HPV-related cancers. In addition, attitudes toward HPV vaccination were assessed only among female participants, in line with the expected target population of the future vaccination program. Consequently, our findings cannot be generalized to males, despite the fact that HPV vaccination also protects against several other HPV-related cancers affecting both sexes.

## 5. Conclusions

In conclusion, this study provides data regarding CC and HPV awareness and attitudes toward HPV vaccines. The results show that despite the high level of awareness about CC, an information vacuum remains, represented by the low rate of awareness of HPV and a medium level of knowledge regarding HPV importance, epidemiology, and prevention. These results were associated with a low level of vaccine acceptance and adherence to CC screening. Thus, increasing awareness about HPV and its public health importance among the Algerian population is the first step that should be engaged in the efforts of prevention that may certainly help to convince the population about the importance of HPV vaccines and consequently increasing its acceptance.

## Figures and Tables

**Table 1:** Participant sociodemographic characteristics.

Variable	Outcome	Number	Percentage (%)
Age	18–30 years old	309	74.6
	Over 30 years old	105	25.4
Educational level	No university education	100	24.2
	University education	314	75.8
Gender	Female	154	37.2
	Male	260	62.8
Marital status	Single	297	71.7
	Married (divorced, widowed)	117	28.3
Residence	Rural	29	7.0
	Urban	385	93.0
Financial level	Low	47	11.4
	Medium	321	77.5
	High	46	11.1
Healthcare worker	No	351	84.8
	Yes	63	15.2

**Table 2:** Regression analysis of the factors associated with CC awareness.

Variables	B	SE	Wald	Sig.	OR	95% CI OR	
						Lower	Upper
Age [>30 years old]	1.022	0.383	7.142	**0.008**	2.78	1.313	5.883
Age [≤30 years old]	Ref.	.	.	.	.	.	.
Gender [Female]	0.703	0.287	5.978	**0.014**	2.02	1.15	3.548
Gender [Male]	Ref.	.	.	.	.	.	.
Marital status [Single]	−0.435	0.346	1.581	0.209	0.647	0.328	1.276
Marital status [Married]	Ref.	.	.	.	.	.	.
Financial level [High]	0.516	0.495	1.085	0.298	1.675	0.635	4.419
Financial level [Medium]	0.812	0.343	5.596	**0.018**	2.252	1.149	4.413
Financial level [Low]	Ref.	.	.	.	.	.	.
Healthcare worker [Yes]	2.839	1.025	7.672	**0.006**	17.099	2.294	127.476
Healthcare worker [No]	Ref.	.	.	.	.	.	.

Bold characters indicate significant results with a level of significance sig. ≤ 0.05.

**Table 3:** Regression analysis of the factors associated with HPV awareness.

Variables	B	SE	Wald	Sig.	OR	95% CI OR	
						Lower	Upper
Age [>30 years old]	−0.777	0.274	8.051	**0.005**	2.175	1.272	3.719
Age [≤30 years old]	Ref.	.	.	.	.	.	.
Gender [Male]	0.099	0.274	0.132	0.716	1.105	0.646	1.889
Gender [Female]	Ref.	.	.	.	.	.	.
Financial level [High]	1.16	0.515	5.069	**0.024**	3.189	1.162	8.751
Financial level [Medium]	0.39	0.421	0.858	0.354	1.478	0.647	3.375
Financial level [Low]	Ref.	.	.	.	.	.	.
Healthcare worker [Yes]	−2.533	0.363	48.612	**<0.001**	12.591	6.177	25.661
Healthcare worker [No]	Ref.	.	.	.	.	.	.

Bold characters indicate significant results with a level of significance of sig. ≤ 0.05.

**Table 4:** Score of knowledge about HPV among the study population.

Variable	Outcome	Mean	SE	Sig.
Age	18–30 years old	6.64	2.806	0.456
	Over 30 years old	6.33	2.909	
Educational level	No university education	6.83	2.806	**0.023**
	University education	5.46	2.715	
Gender	Female	6.77	2.807	0.408
	Male	6.36	2.86	
Marital status	Single	6.4	2.981	0.416
	Married	6.82	2.525	
Residence	Rural	3	1.732	**0.04**
	Urban	6.68	2.781	
Financial level	Low	7.35	2.948	NS
	Medium	6.32	2.832	
	High	7	2.268	
Healthcare worker	No	5.58	2.581	<0.001
	Yes	8.14	2.507	

Bold characters indicate significant results with a level of significance of sig. ≤ 0.05. NS: not significant.

**Table 5:** Attitudes toward HPV vaccines and barriers leading to reluctance.

Items	Number	Percentage (%)
**Got HPV Vaccine**		
Yes	3	2.3
No	130	97.7
**Intention to get the vaccine**		
Yes	40	30.8
No	36	27.7
IDK	54	41.5
**Cause of vaccine reluctance**		
I do not consider myself at risk of HPV infection	22	25.6
I do not consider HPV as a common infection in Algeria	15	17.4
Visiting a doctor makes me uncomfortable, which keep me from vaccination	11	12.8
I am against vaccines in general	9	10.5
I consider these vaccines ineffective	8	9.3
I consider these vaccines unsafe	6	7.0
I am not completely against these vaccines/I will think about them	4	4.7
I do not feel well after being vaccinated	2	2.3
Others	9	10.5

**Table 6:** Relationship between attitudes toward HPV vaccine and knowledge and conspiracy belief scores.

Score		Attitude Toward HPV Vaccine			Total
		Willing (W)	Reluctant (R)	Hesitant (H)	
VCBS	Mean	19.78	25.14	22.17	22.25
	SE	8.954	10.45	9.65	9.816
	Sig.	**0.045 (W vs. R)**	0.330 (R vs. H)	0.464 (W vs. H)	.
Knowledge	Mean	8.02	5.22	6.24	6.54
	SE	2.209	3.034	2.642	2.832
	Sig.	**0.000 (W vs. R)**	**0.0173 (R vs. H)**	**0.003 (W vs. H)**	.

H: hesitant, R: reluctant, W: willing. Bold characters indicate significant results with a level of significance of sig. ≤ 0.05.

## Data Availability

The data that support the findings of this study are available from the corresponding author upon reasonable request.

## Supplementary Materials

The following supporting information can be downloaded at https://www.mdpi.com/article/10.3390/vaccines14080676/s1; Table S1: Factors influencing CC awareness; Table S2: Factors influencing HPV awareness; Questionnaire S1: Awareness and Knowledge about Cervical Cancer and HPV in Algeria.

## Abbreviations

The following abbreviations are used in this manuscript:

CC	Cervical Cancer
HPV	Human Papillomavirus
WHO	World Health Organization
