# Supplementary material for: Awareness and Knowledge Levels of Cervical Cancer and HPV and Attitudes Toward HPV Vaccination in Algeria
Source: Vaccines (Basel). 2026 Aug 5;14(8):676. doi: 10.3390/vaccines14080676 (PMC13517512; doi:10.3390/vaccines14080676)
Supplement: Supplementary file 1 [file vaccines-14-00676-s001.zip › vaccines-4454841-supplementary.pdf]

Supplementary materials

Awareness and Knowledge about Cervical Cancer and HPV in Algeria

Table S1. Factors influencing CC awareness

Variables	Outcomes	CC awareness		Sig.
		No	Yes	
		Number (%)	Number (%)	
**Age**	18-30 years old	83 (26.9)	226 (73.1)	**0.002**
	Over 30 years old	13 (12.4)	92 (87.6)	
**Educational level**	No university education	27 (27.0)	73 (73.0)	0.3
	University education	19 (22.4	66 (77.6)	
**Gender**	Female	23 (14.9)	131 (85.1)	**0.002**
	Male	73 (28.1)	187 (71.9)	
**Marital status**	Single	78 (26.3)	219 (73.7)	**0.018**
	Married	18 (15.4)	99 (84.6)	
**Residence**	Rural	11 (37.9)	18 (62.1)	0.051
	Urban	85 (22.1)	300 (77.9)	
**Financial level**	Low	20 (42.6)	27 (57.4)	**0.004**
	Medium	66 (20.6)	255 (79.4)	
	High	10 (21.7)	36 (78.3)	
**Healthcare worker**	No	95 (27.1)	256 (72.9)	**<0.001**
	Yes	1 (1.6)	62 (98.4)	

Bold character indicated significant results with a level of significance Sig.≤0.05

Tables S2. Factors influencing HPV awareness

Variables		HPV Awareness		Sig.
		No	Yes	
		Number (%)	Number (%)	
**Age**	18-30 years old	219 (70.9)	90 (29.1)	**0.025**
	Over 30 years old	62 (59.0)	43 (41.0)	
**Educational level**	No university education	72 (72.0)	28 (28.0)	0.31
	University education	209 (66.6)	105 (33.4)	
**Gender**	Female	94 (61.0	60 (39.0)	**0.022**
	Male	187 (71.9)	73 (28.1)	
**Marital status**	Single	209 (70.4)	88 (29.6)	0.083
	Married	72 (61.5)	45 (38.5)	
**Residence**	Rural	24 (82.8)	5 (17.2)	0.075
	Urban	257 (66.8)	128 (33.2)	
**Financial level**	Low	39 (83.0)	8 (17.0)	**0.003**
	Medium	219 (68.2)	102 (31.8)	
	High	23 (50.0)	23 (50.0)	
**Healthcare worker**	No	268 (76.4)	83 (23.6)	**<0.001**
	Yes	13 (20.6)	50 (79.4)	

Bold character indicated significant results with a level of significance Sig.≤0.05

Questionnaire S1

Awareness and Knowledge about Cervical Cancer and HPV in Algeria

Q1- Have you heard of human papillomavirus HPV?

Q2- If you answered yes, what is/are your main source of knowledge about HPV?

Q3- Do you know HPV induced diseases?

Q4- If you answered yes, which are them?

Q5- Have you heard of cervical cancer?

Q6- If you answered yes,what is/are yourmain source of knowledge about cervical cancer?

Q7- If you answered yes, do you know the causes of cervical cancer?

Q8- If you answered yes, what are the causes of cervical cancer?

Q9- Please answer the following questions based on your knowledge prior to this survey

**Item**	**YES**	**NO**
**1.** HPV infections are rare	◎	◎
**2.** HPV is sexually transmitted	◎	◎
**3.** Genital warts are caused by HPV	◎	◎
**4.** HPV can cause cervical cancer	◎	◎
**5.** Men cannot get HPV	◎	◎
**6.** Individuals can be infected by HPV for years without knowing	◎	◎
**7.** HPV can be cured by antibiotics	◎	◎
**8.** Are you aware of the availability of HPV vaccines?	◎	◎

Q10- Are you aware of the availability of cervical cancer screening test?

Q11- If you answered yes, what is the cervical cancer screening test?

Q12- Have you ever had a cervical cancer screening test (for women)?

Q13- If you didn't do early cervical cancer screening, what is/are principal reason(s)?

Q14- Are you aware of the availability of HPV vaccines?

Q15- Given that HPV vaccines are safe and effective to protect from infection by the most common HPV types associated with cervical cancer:

1. Have you received HPV vaccination?

2. Are you willing to get HPV vaccine if provided for free?

3. If you answered no,what is/are themain reason(s) for your refusal to getHPV vaccine?

4. Are you willing to pay to get HPV vaccine?

5. What was your main source of knowledge about HPV?

Q16- For each statement, please indicate how much you disagree or agree by selecting the appropriate number:

	Strongly Disagree	*Disagree*	*Somewhat Disagree*	*Neutral*	*Somewhat Agree*	*Agree*	*Strongly Agree*
**1.** Vaccine safety data is often fabricated.	1	2	3	4	5	6	7
**2.** Immunizing children is harmful, and this fact is covered up.	1	2	3	4	5	6	7
**3.** Pharmaceutical companies cover up the dangers of vaccines.	1	2	3	4	5	6	7
**4.** People are deceived about vaccine efficacy.	1	2	3	4	5	6	7
**5.** Vaccine efficacy data is often fabricated.	1	2	3	4	5	6	7
**6.** People are deceived about vaccine safety.	1	2	3	4	5	6	7
**7.** The government is trying to cover up the link between vaccines and autism.	1	2	3	4	5	6	7
